# Children’s Drawings as a Tool to Explore the Emotional Experience of Migrant Children in Dental Care: A Qualitative Study in Italy

**DOI:** 10.3390/children13040468

**Published:** 2026-03-28

**Authors:** Lucia Giannini, Chiara Alessandra Dini, Gregorio Menozzi, Maria Assunta Mauri, Federica Macrì, Ioana Roxana Bordea, Francesca Calò, Lucia Memè, Andrea Palermo

**Affiliations:** 1 Fondazione IRCCS Cà Granda Ospedale Maggiore Policlinico, 20122 Milan, Italy; 2 Department of Biomedical, Surgical and Dental Sciences, University of Milan, 20122 Milan, Italy; 3 Department of Oral Rehabilitation, Faculty of Dentistry, Iuliu Hațieganu University of Medicine and Pharmacy, 400012 Cluj-Napoca, Romania; 4 Department of Interdisciplinary Medicine, University of Bari “Aldo Moro”, 70121 Bari, Italy; 5 Department of Life Sciences, Health and Health Professions, Link Campus University Rome, 00165 Rome, Italy; 6 Department of Experimental Medicine, University of Salento, 73100 Lecce, Italy

**Keywords:** communication barriers, dental anxiety, orthodontics, pediatric dentistry, transients and migrants

## Abstract

**Highlights:**

**What are the main findings?**

- Migrant children’s drawings during dental visits revealed clear emotional markers of anxiety and perceived threat, with differences according to age and drawing development.
- Drawing emerged as a valuable non-verbal clinical and communicative tool to access children’s emotional experience despite language barriers.

**What are the implications of the main findings?**

- Understanding emotional responses through drawings can help clinicians to identify anxiety and vulnerability early and tailor their approach during dental treatment.
- The findings support the use of culturally sensitive communication strategies, including visual aids and multilingual materials, to improve cooperation and treatment outcomes in migrant pediatric patients.

**Abstract:**

**Background**: In multicultural healthcare systems such as the Italian one, migrant children may experience dental care as particularly stressful because linguistic and cultural barriers can limit communication, emotional expression, and understanding of the clinical setting. **Aim**: Understanding the emotional experience of migrant children during dental visits is essential for improving clinical management in pediatric dentistry and orthodontics within multicultural contexts. Because linguistic barriers often limit verbal communication, this study aimed to explore children’s mental representations, emotional states, and perceptions of the dental environment through drawing and to evaluate the clinical implications for communication and therapeutic collaboration. **Methods**: This qualitative study was conducted in Italy between 2016 and 2025 and analyzed 50 drawings produced by 50 foreign-born migrant children aged 6–13 years, recruited through an educational cooperative in Piacenza. Most participants originated from developing countries and had limited proficiency in Italian, frequently showing a marked “experience gap” in drawing ability that interfered with normative developmental stages described by Lowenfeld. The analysis focused on spatial organization, line quality, color use, posture, interpersonal distance, and representation of the clinical environment, integrating graphic competence assessment with emotional interpretation. **Results**: Younger children commonly depicted rigid lines, essential settings, and oversized dental unit lamps, whereas older children increasingly represented threatening or disproportionate instruments, aggressive dentists, and omission of the patient figure. Around age 10, drawings became more detailed and colorful, although symbols of closure, such as locked doors, persisted. In adolescents, representations polarized between rich, coherent scenes and extremely essential drawings dominated by fear, rigidity, minimal environments, and symbols of constraint. The findings suggest that drawing may represent a valuable non-verbal clinical and communicative resource for exploring migrant children’s emotional experience of dental care and for identifying signs of anxiety and vulnerability that may not emerge through verbal interaction alone. **Conclusions**: These findings support the value of a culturally sensitive dental approach integrating drawing, visual aids, multilingual educational materials, and play-based strategies to reduce communication barriers and improve cooperation in migrant children receiving pediatric dental and orthodontic care.

## 1. Introduction

Health protection represents a fundamental right of the individual and an interest of the community, as established by Article 32 of the Italian Constitution. In recent decades, Italy has experienced a steady increase in the foreign resident population, characterized by a younger age distribution compared to Italian nationals and by a growing proportion of children. According to the most recent ISTAT estimates (2024–2025), foreign residents account for more than 10% of the total population, with more than one million minors and a significant number of second-generation individuals [1,2,3,4,5,6,7,8]. In addition, the presence of undocumented migrants whose number cannot be fully quantified further complicates the assessment of healthcare needs.

Italian legislation ensures full access to essential and urgent healthcare services for both regular and irregular migrants [9,10,11,12,13,14,15,16,17]. Consequently, the healthcare system, including dentistry, increasingly encounters patients from highly heterogeneous cultural, linguistic, and healthcare backgrounds. This diversity is associated with communication barriers, difficulties in symptom interpretation, divergent perceptions of illness, and linguistic obstacles that may negatively influence the quality of care [18,19,20,21,22].

The situation is particularly delicate for migrant children. Cultural barriers combine with age-related fears, limited ability to verbalize emotional experiences, and difficulty understanding complex clinical explanations [23,24,25,26,27,28,29,30,31,32,33,34,35]. Moreover, many children may originate from countries marked by social instability, poverty, or traumatic healthcare experiences, contributing to heightened vulnerability and an increased prevalence of dental fear/anxiety [36,37,38,39,40,41,42,43,44].

Dental visits are frequently perceived by children as potentially stressful experiences. In vulnerable pediatric patients, the use of minimally invasive and better-tolerated treatment options, such as silver diamine fluoride and atraumatic restorative approaches for primary teeth, may help reduce the emotional burden of care and improve treatment acceptance [34,44]. Previous studies have shown that dental fear and anxiety are among the most common emotional reactions in pediatric dental settings and may arise from several factors, including fear of pain, unfamiliar instruments, loss of control, and the sensory characteristics of the clinical environment. These emotional responses can influence children’s behavior during treatment, their level of cooperation with healthcare professionals, and their willingness to attend dental appointments.

These challenges may be even more pronounced in migrant children, who may experience additional barriers such as linguistic difficulties, cultural differences in the perception of healthcare, and limited familiarity with preventive dental care practices. Understanding how children perceive and mentally represent the dental environment is therefore important for improving communication strategies, facilitating adaptation to clinical settings, and promoting more effective and culturally sensitive approaches in pediatric dental care.

Children’s drawings represent a privileged and universally accessible communicative tool. Drawing enables children to express perceptions, fears, memories, and internal representations even in the absence of adequate linguistic skills. For this reason, drawings have long been used not only in psychoeducational contexts but also in psychological assessment and developmental research as a means of exploring children’s emotional experiences and representations of complex situations. In recent years, the literature has increasingly emphasized the role of children’s drawings as a valuable methodological tool for accessing children’s subjective perspectives and emotional meanings, particularly in contexts where verbal communication is limited or culturally mediated.

Owing to its symbolic and emotional value, drawing can act as a crucial communicative bridge between migrant children and healthcare providers, facilitating understanding of their emotional experience of dental care [45]. At the same time, healthcare professionals play a key role in transcultural communication. They are required to adapt language, educational methods, and motivational strategies, relying on visual tools, multilingual material, and an empathetic relational approach to enhance compliance and reduce barriers [46,47,48,49,50,51].

Although the present study is primarily grounded in pediatric dentistry, its implications may also extend to orthodontics, where treatment success often depends on repeated visits, clear communication, home-care instructions, and long-term cooperation from both the child and caregivers. This is particularly relevant in preventive and interceptive care, where repeated attendance, understandable instructions, and sustained family involvement are essential for early caries management and for orthodontic treatments in growing patients, including clear-aligner protocols [6,8]. In this context, understanding children’s emotional representations of dental care may help clinicians identify barriers to adherence early and adapt their communicative and relational strategies accordingly.

Children’s drawings have long been considered a valuable non-verbal resource for accessing children’s subjective experiences, particularly when verbal expression is limited by age, emotional distress, or linguistic barriers. In healthcare settings, drawings have been used to explore children’s perceptions, fears, and emotional responses to clinical environments. In pediatric dentistry specifically, previous research has shown that drawings may help identify pain, fear, anxiety, stress/distress, and children’s perceptions of the dentist and dental treatment. For example, previous studies have used children’s drawings to better understand experiences of dental treatment under general anesthesia and as a non-verbal measure of dental anxiety. A recent scoping review further concluded that children’s drawings can be a useful tool for identifying emotional responses and perceptions related to dental care.

Against this background, the present study aims to analyze drawings produced by 50 migrant children from developing countries in order to explore how they experience, imagine, or recall dental care, as well as to identify recurring emotional patterns relevant to intercultural communication and clinical practice [52,53,54,55,56,57,58,59,60,61,62]. Although previous studies have used children’s drawings to explore dental anxiety, perceptions of the dentist, and experiences of dental treatment, relatively few studies have specifically focused on foreign-born migrant children in routine dental care and interpreted these representations from a transcultural communication perspective. The present study was designed to contribute to this emerging area by analyzing how migrant children emotionally represent dental care and by discussing the implications for culturally sensitive clinical communication [27,63,64,65,66,67,68,69,70,71,72,73,74,75]. The present study was designed to contribute to this more specific area by analyzing how migrant children emotionally represent dental care and by discussing the implications for culturally sensitive clinical communication. 

## 2. Materials and Methods

### 2.1. Study Design and Setting

This observational qualitative study was based on the analysis of children’s drawings. The study was conducted between 2016 and 2025 and involved the collection and analysis of 50 drawings produced by 50 foreign-born children aged 6–13 years. This extended timeframe was chosen to capture a broad range of migratory backgrounds and social contexts potentially influencing the perception of oral healthcare. The sample was obtained through collaboration with the Cooperativa Sociale “Mondo Aperto” (Piacenza, Italy), an organization engaged in literacy and integration programs for migrant families.

Although the drawings were collected over a long period (2016–2025), the aim of the study was not to examine temporal trends but to identify stable emotional and symbolic patterns in migrant children’s representations of dental care. Therefore, drawings from different years were pooled into a single qualitative dataset and analyzed collectively.

### 2.2. Participants and Sample Characteristics

All participating children were born abroad and later migrated to Italy to join or reunite with their parents. Their developmental trajectories therefore unfolded within cultural, social, economic, and healthcare environments markedly different from the Italian context. Most children originated from developing countries characterized by limited access to healthcare systems and a poorly established culture of prevention. None of the participants spoke Italian as their mother tongue, and many demonstrated limited oral and written proficiency.

The inclusion criteria were designed to focus on children with significant linguistic barriers, making drawing a primary and universal communicative bridge capable of bypassing the limitations of traditional verbal anamnesis in transcultural settings.

### 2.3. Ethical Considerations

The study was conducted in accordance with the principles of the Declaration of Helsinki. Written informed consent was obtained from the parents or legal guardians of all participating children. The drawings were collected within educational and clinical–relational activities, and all data were fully anonymized. The study protocol was approved by the Ethics Committee (protocol code 421; date of approval: 9 March 2016).

### 2.4. Theoretical Framework

Lowenfeld’s developmental model, one of the most authoritative frameworks for understanding children’s graphic development, describes drawing evolution through progressive stages, namely scribbling, pre-schematic, schematic, dawning realism, and pseudo-realism, each associated with specific cognitive, motor, symbolic, and emotional competencies.

However, when applied in multicultural contexts, this model shows important limitations, particularly in children from developing countries or from environments lacking graphic-expressive stimulation. In the present sample, a marked “experience gap,” related to limited exposure to drawing materials, inadequate schooling, or fragmented educational histories, resulted in delays or deviations from the developmental sequence described by Lowenfeld. This “experience gap” was therefore interpreted not as a developmental deficit, but as a sociocultural variable relevant to the interpretation of the drawings.

### 2.5. Administration of the Drawing Activity

The drawing activity was administered and collected by a dentist and a pedagogist with experience in working with children in educational contexts. Both provided standardized instructions to the participants and ensured that the activity was carried out in a calm and supportive environment suitable for children. The drawings were subsequently analyzed according to the predefined indicators by the research team. The children were individually invited to produce a drawing during educational activities organized by the cooperative. Each child was provided with blank paper and colored pencils. The instruction given was simple and open-ended: the children were asked to “draw what comes to mind when you think about going to the dentist” or “draw a dental visit.” This formulation was intentionally chosen to allow children to freely express their perceptions and experiences without imposing a predefined structure. The clinician was present during the activity but did not interfere with the child’s production, except for giving the initial instructions and providing practical support when necessary. The drawing activity took place in a familiar educational environment and was presented as a playful and voluntary activity. No time limits were imposed, and children were encouraged to describe their drawing if they wished.

### 2.6. Qualitative Analysis of Drawings

The qualitative analysis was conducted through a dual interpretative framework combining formal graphic observation with symbolic–relational content analysis. This approach was chosen in order to capture both the child’s level of graphic-expressive organization and the emotional meaning attributed to the dental experience.

In a first analytical step, each drawing was examined for formal and structural indicators, including spatial organization, the size and proportion of figures, line pressure and quality, use of color, level of detail, erasures, symmetry or asymmetry, and signs of movement or rigidity. These elements were considered useful for describing the child’s graphic competence and the degree of tension, control, or inhibition expressed in the drawing.

In a second step, symbolic–relational content was explored. Particular attention was paid to the representation of roles within the scene (dentist, assistant, patient, parent), interpersonal distance, body posture, facial expression, and the depiction of the clinical environment, including the dental chair, lamp, instruments, windows, and doors. Recurrent visual elements such as oversized lights, sharp or disproportionate instruments, interposed objects, closed doors, absent patient figures, rigid postures, and darkened areas were interpreted as possible indicators of perceived threat, passivity, vulnerability, avoidance, relational distance, or loss of control.

The analysis followed an iterative and inductive process. After an initial open reading of all drawings, recurrent visual and symbolic elements were identified and progressively grouped into broader interpretative categories reflecting emotional experience in the dental setting. The final categories were derived from repeated patterns observed across the sample and were supported, when available, by children’s spontaneous verbalizations produced during or immediately after the drawing activity. These verbalizations were not treated as an independent dataset, but as contextual material useful for strengthening the plausibility of the graphic interpretation.

### 2.7. Evaluator Agreement and Interpretive Consistency

All drawings were independently reviewed by two designated evaluators with specific experience in pediatric clinical settings and in the qualitative interpretation of children’s drawings from a developmental and relational perspective. Not all co-authors were involved in this interpretative phase; the multidisciplinary authorship of the manuscript reflects the broader clinical and academic contribution to the study, whereas the graphic analysis was performed by the designated evaluators.

This consensus-based procedure was adopted to reduce individual interpretative bias and to enhance the internal consistency of the analysis.

To further improve methodological rigor, the interpretation of the drawings was grounded in repeated comparison between formal graphic features, symbolic content, and recurring cross-case patterns. The use of this triangulated and consensus-based qualitative procedure supported the reproducibility and credibility of the findings, while preserving the exploratory nature of the study. The graphic indicators used in the present study were derived from established frameworks in the literature on children’s graphic development and emotional expression in drawings. In particular, the developmental model proposed by Lowenfeld provides a widely recognized reference for understanding the relationship between drawing features and children’s cognitive and emotional development. Additional studies on children’s drawings and projective graphic indicators have also highlighted how elements such as line pressure, spatial organization, figure proportions, and symbolic objects may reflect emotional states such as tension, fear, or vulnerability.

## 3. Results

The analysis of the 50 collected drawings showed that drawing constitutes an effective expressive medium for accessing the emotional experience of migrant children during dental visits. Despite substantial linguistic barriers, children were able to depict emotions, perceptions, and symbolic elements associated with the dentist and the clinical environment. The presence of 22 spontaneous verbalizations further enriched the interpretation, allowing specific graphic elements to be linked to explicitly expressed emotional content. These verbalizations acted as a qualitative support for the interpretation, confirming that graphic indicators of tension such as heavy strokes, angular lines, and frequent erasures corresponded to explicitly stated feelings of fear, discomfort, or vulnerability.

### 3.1. Age-Related Graphic and Emotional Patterns

Age-based analysis revealed considerable variability in expressive abilities, together with recurring emotional and symbolic elements throughout development (Table 1).

In the drawings of 6 and 7 year old children, angular lines, rigid figures, and sparse or absent background settings predominated. The dental lamp appeared frequently, often enlarged beyond realistic proportions, as if representing a central and disturbing element of the dental experience. Objects placed between dentist and patient, as well as closed doors or windows, emerged even at this age as symbols of distance, protection, or absence of escape routes. Dark and intense colors, particularly black, red, and deep blue, were common. Facial expressions, when present, ranged from neutral to forced smiles, with graphic indicators of tension such as spiky hair or stiff, immobile postures.

Among 8 and 9 year old children, lines remained heavy, and the depiction of the dental office continued to be minimal or symbolic; however, the number of instrument-related details increased, often appearing disproportionate, oversized, or pointed. Emphasis on instruments, especially syringes, drills, and cutting tools, suggested increasing awareness of invasive dental procedures. In this age group, explicit verbalizations of fear, pain, or requests for help became more frequent, and patient postures were almost always static and rigid, sometimes portrayed as lying on an operating table, highlighting a perception of passivity and vulnerability.

Around age 10, greater use of color and more elaborate backgrounds appeared, particularly in girls’ drawings, which included furniture, sinks, cabinets, and even comforting objects such as stuffed animals. Nonetheless, the enlarged dental lamp and the recurrent presence of closed doors persisted as symbolic indicators of discomfort and closure. Verbalizations included both reassuring phrases attributed to the dentist and explicit expressions of fear. Boys’ drawings at the same age showed poorer or incongruent settings, with dental chairs placed outdoors or in undefined spaces, indicating uneven graphic development and a fragmented perception of the clinical context.

In drawings by 11 and 12 year old children, more complex and symbolically rich elements emerged, including dentists depicted as threatening figures, coats resembling butcher aprons, exaggerated pointed instruments, and, in boys, the complete absence of the patient. These representations suggested a combination of fear and projected aggression toward the dentist, sometimes accompanied by dramatic or alarmed verbalizations. Girls’ drawings, although more orderly and linear, continued to portray enclosed environments and rigid postures, occasionally associated with expressions of pain or crying.

At age 13, a stronger polarization was observed: some drawings were structured and coherent, whereas others were extremely essential, lacking background details and featuring incomplete or stylized human figures. Closed doors continued to appear systematically, as did the oversized dental lamp, often drawn so large as to dominate the scene. Boys’ drawings were particularly simplified or distorted, with dentists lacking proportion or rendered as ghost-like figures. Pointed instruments and verbalizations of pain or fear persisted.

**Figure 1:**
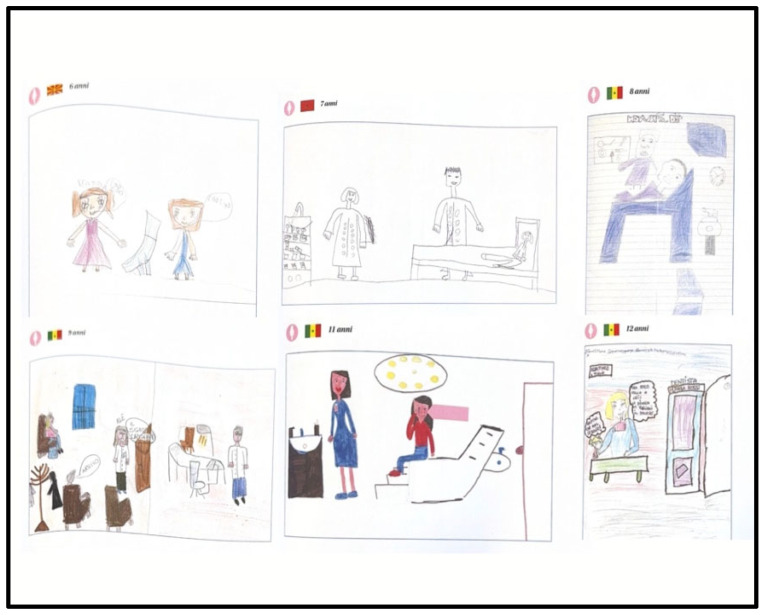
Six drawings by girls aged 6–12 from various migratory backgrounds, illustrating access and presence in the dental clinic, with increasing detail in the setting and relational dynamics.

**Figure 2:**
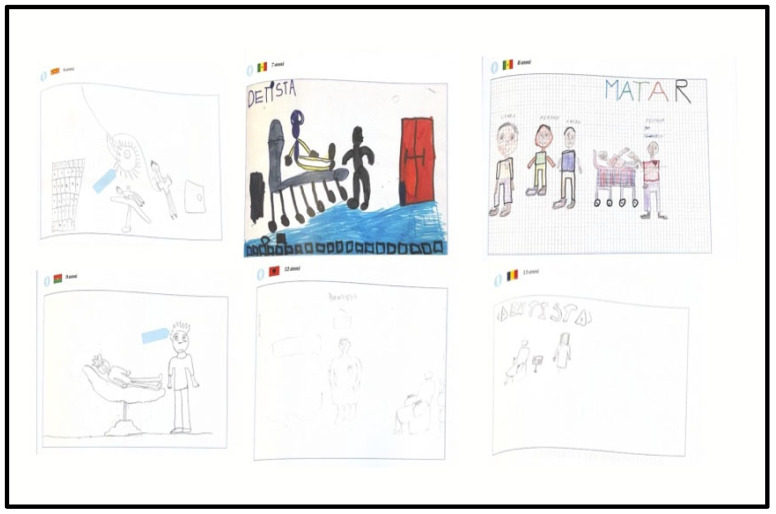
Six drawings by boys aged 6–13 showing enlarged dental lights, emphasized instruments, rigid or supine patients, and interposed objects, reflecting fear, vulnerability, and emotional distance.

Figure 1 and Figure 2 present a representative selection of drawings grouped by age and sex, illustrating the main graphic and emotional patterns identified across the sample.

### 3.2. Recurrent Symbolic Indicators of Anxiety and Vulnerability

Qualitative analysis additionally revealed a set of symbolic recurrences which, regardless of age or geographical origin, appeared with high frequency and semantic coherence. These graphic elements represent meaningful indicators of the child’s emotional experience in the dental context and allow the identification of stable patterns of anxiety, vulnerability, and perceived threat.

Recurrent elements such as the oversized dental lamp, disproportionate instruments, closed doors, rigid postures, absent patient figures, and motionless bodies represented as if lying on an operating table formed a coherent picture of fear, passivity, loss of control, and need for protection. Differences in graphic maturity were largely linked to limited access to early graphic-expressive experiences rather than to developmental deficits. Table 2 summarizes the main recurrent symbols and their clinical–emotional interpretation.

### 3.3. Spontaneous Verbalizations Associated with the Drawings

The spontaneous verbalizations collected during or immediately after the drawing activity provided useful contextual support for interpreting the visual material. Expressions such as requests for help, statements of fear, references to pain, and reassuring phrases attributed to the dentist helped clarify the emotional meaning of several graphic features. Although limited in number, these verbalizations reinforced the interpretation of the drawings as reflecting a complex emotional experience often marked by anxiety, distrust, passivity, and, in some cases, projected aggressiveness.

## 4. Discussion

The present findings suggest that children often represent dental care through visual markers of fear, passivity, and perceived threat [76,77,78,79,80,81,82,83,84,85,86].

These representations are clinically relevant because they may reveal emotional barriers that are not easily accessible through verbal communication alone, especially in children with limited proficiency in the host country’s language [87,88,89,90,91,92,93].

From a clinical perspective, this has implications not only for pediatric dentistry but also for orthodontics. This also applies to developmental and functional conditions that require timely diagnosis, repeated monitoring, and clear communication with families, such as Class II functional treatment, occlusal-functional assessment, and the management of infra-occluded deciduous molars [94,95,96]. In both settings, successful care depends on trust, repeated attendance, comprehension of instructions, and cooperation over time. This is particularly relevant in orthodontics, where treatment frequently requires continuity, home-care adherence, and sustained engagement by both the child and caregivers. In this context, understanding the child’s emotional representation of dental care may help clinicians prevent disengagement and tailor communication strategies from the earliest stages of treatment.

The analysis of drawings produced by migrant children revealed that their emotional experience of dental care is profoundly shaped by multiple factors, including previous experiences, cultural background, linguistic barriers, familiarity with healthcare environments, and degree of social integration [97,98,99]. This aligns with evidence showing higher susceptibility to dental fear and anxiety (DFA) in children belonging to ethnic minorities or migrant families [100,101,102,103]. Linguistic difficulties and limited exposure to structured healthcare settings represent critical obstacles, often compounded by previous traumatic experiences that may heighten negative emotional reactions [104,105,106,107].

When fear and poor cooperation become clinically significant, the management of pediatric dental anxiety may also require the integration of behavioral, iatrosedative, or pharmacological strategies, especially in particularly fragile children [80,81,82,83,84,85,86,87,88,89,90,91,92,93,94,95,96,97,98,99,100,101,102,103,104,105,106,107,108]. In this context, drawing appeared to function as a useful non-verbal medium for accessing children’s emotional representations when verbal communication was limited [109,110,111,112]. In dental settings, drawings allow clinicians to access not only perceptions of pain or fear but also the child’s internal representations of the dentist, instruments, and clinical environment.

Recurrent graphic elements such as oversized dental lights, disproportionate instruments, rigid body postures, and closed doors carry clear symbolic meaning, representing feelings of entrapment, vulnerability, threat, or lack of control. Notably, the presence of aggressive content in drawings by children from Eastern Europe is consistent with studies on the psychological impact of migration from areas affected by high social stress or conflict [113,114,115,116,117,118,119,120,121,122,123,124,125,126,127,128].

These findings highlight the need to move beyond a purely technical conception of dental visits toward a biopsychosocial and culturally sensitive model of care. Since the results highlight a recurrent perception of the dentist as a threatening or punitive figure (e.g., ‘butcher-like’ attire or oversized instruments), the integration of play-based strategies becomes a clinical necessity to reshape this mental representation and foster a trust based relationship. Numerous authors have emphasized that the effectiveness of dental treatment in migrant children depends largely on the clinician’s ability to establish an empathetic, comprehensible, and reassuring relationship capable of bridging linguistic and cultural barriers [129,130,131].

**Figure 3:**
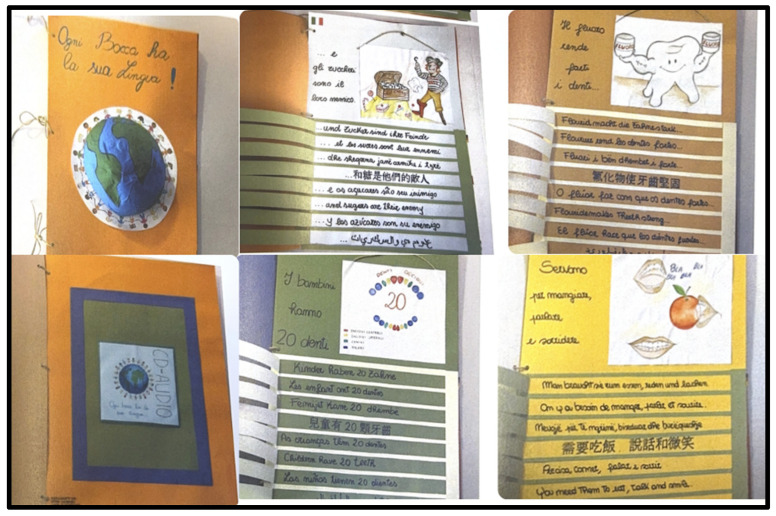
Example of multilingual illustrated oral-health material designed to support communication with migrant children and families.

Clinical experience in pediatric dentistry and orthodontics confirms that obtaining collaboration from migrant children can be particularly challenging when adequate communication tools are lacking [132,133,134]. Orthodontic treatments requiring high continuity and home care responsibilities tend to show reduced access and lower completion rates among migrant youth compared with native peers [135,136,137] (Figure 3).

**Figure 4:**
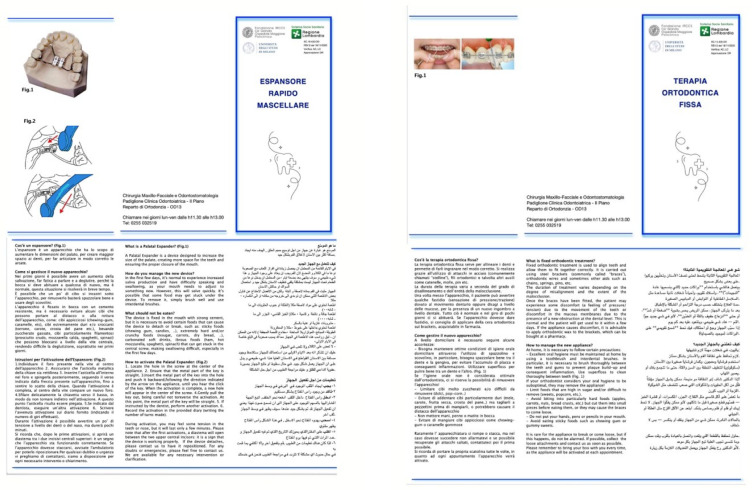
Example of multilingual orthodontic information material designed to support communication with migrant patients and caregivers.

To exemplify the type of culturally sensitive communication tools that may support clinical practice, two illustrative examples of multilingual educational materials are presented below. These materials are included for explanatory purposes and do not form part of the analyzed dataset (Figure 4). These tools help enhance therapeutic adherence, reduce communication misunderstandings, and improve the overall effectiveness of treatment [138,139].

In summary, this study not only deepens the understanding of migrant children’s emotional experiences but also provides concrete operational indications for improving daily clinical practice [138,139,140,141,142,143,144,145,146,147].

These strategies help to reduce fear, enhance cooperation, and improve the success of pediatric and orthodontic treatments, ultimately contributing to better oral health and overall well-being among migrant children [148,149,150]. These findings support the relevance of culturally sensitive communication in pediatric dental care. In clinical practice, drawing may be considered a useful non-verbal relational resource, particularly during the first contact with migrant children, as it may help clinicians identify fear, vulnerability, and relational distance that are not easily accessible through verbal interaction alone. The use of visual aids, multilingual materials, and play-based communication strategies may further facilitate understanding, reduce perceived threat, and support cooperation in both pediatric dentistry and, more cautiously, in orthodontic care when long-term adherence is required [5,151,152,153,154,155,156,157,158]. 

### 4.1. Clinical Recommendations

The findings suggest that drawing may be considered as a useful clinical–relational resource during the first dental visits of migrant children. It may help clinicians identify early signs of anxiety, fear, and vulnerability that do not easily emerge through verbal communication alone. Based on the observed graphic patterns, clinicians may consider the use of visual explanations, multilingual educational materials, and play-based communication strategies to reduce perceived threat and increase the child’s sense of control. Attention to recurring symbolic indicators, such as oversized dental lights, rigid postures, or closed doors, may also support a more sensitive adaptation of the clinician’s communicative style. In orthodontic settings, where continuity of care and adherence are particularly important, early recognition of emotional barriers may help improve cooperation and family engagement. Tailored communication may be especially relevant in children with chronic or complex clinical conditions, in whom follow-up, multidisciplinary management, and caregiver understanding are important components of treatment quality and long-term adherence [145,147].

### 4.2. Limitation 

This study has several limitations that should be considered when interpreting the findings. First, the sample size was relatively small (50 children, 50 drawings) and was drawn from a specific local context, namely the city of Piacenza and the “Mondo Aperto” Cooperative. This limits the generalizability of the findings to other groups of migrant children with different sociocultural backgrounds or migration histories. In addition, recruitment through a single social cooperative may have introduced selection bias, as participating families were already connected to educational and integration services and may therefore differ from more socially isolated migrant populations. Second, although drawing is a valuable tool for accessing children’s internal representations, both the production and interpretation of drawings involve an unavoidable degree of subjectivity. The absence of a standardized quantitative coding system or psychometric framework may therefore have introduced interpretive variability, despite the use of two independent evaluators.

Another limitation concerns the heterogeneous developmental and educational backgrounds within the sample. Many children showed a marked “experience gap” in drawing, likely related to limited exposure to graphic materials, discontinuous schooling, or reduced opportunities for structured expressive activities. This may have influenced not only the formal quality of the drawings but also the child’s capacity to convey complex symbolic-emotional content. Finally, the absence of a control group of Italian children assessed with the same methodology prevents direct comparison between populations. Future studies including larger samples, quantitative measures, and standardized psychological scales are needed to further explore the clinical applicability of drawing in pediatric dental settings. The present findings should therefore be interpreted with caution and considered exploratory.

## 5. Conclusions

Children’s drawings may offer meaningful insight into how migrant children emotionally experience dental care, making visible fear, vulnerability, and communication barriers that are often difficult to detect through verbal interaction alone. Drawing may therefore represent a useful non-verbal relational resource in pediatric dental care when language barriers limit direct communication. Overall, the findings support the importance of child-centered and culturally sensitive approaches, supported by visual, multilingual, and play-based strategies. Further studies with larger samples and standardized psychological measures are needed to better define the clinical applicability of this approach.

## Figures and Tables

**Table 1:** Summary of age-related graphic features and emotional patterns observed in the drawings.

Age	Graphic Features	Environmental Elements	Representation of the Patient	Emotional and Symbolic Content
6 years	Angular stroke, limited or absent colors, altered proportions	Poor setting, closed doors, enlarged dental light	Rigid, lying, or absent figures; distance from the dentist	Tension (spiky “raised” hair), isolated positive verbalizations, first signs of fear
7 years	Strong stroke, frequent erasures, limited use of colors	Sparse setting, few instruments; enlarged dental light in boys	Rigid, distant patient; in some cases seen from above	Fear, oversized syringes and tools; dentist colored in black in one case
8 years	Angular stroke, minimal use of colors	Clinic depicted as a house; very large dental-unit lights	Patient suffering or shown from behind; rigid, as on an operating table	Verbalizations such as “I’m scared”; sharp tools; spiky “raised” hair
9 years	Rare colors, strong stroke, erasures	Dental light and closed window; teeth and caries shown as dark spots	Rigid or absent patient; dentist shown from behind	Tense expressions, verbalizations such as “help”, menacing figures
10 years	More varied use of colors, greater environmental detail	Closed doors and windows; enlarged dental light	Rigid or frightened patient; sometimes the dental chair appears outdoors	Reassuring or fearful phrases; enlarged tools; presence of stuffed animals
11 years	Almost no color, imprecise stroke	Closed doors, enlarged dental light	Patient crying, rigid, or seen from above	Frightened tone, threatening instruments, disproportionate dentist
12 years	Limited colors, many erasures	Sparse setting, closed door	Patient often absent; static figures	Anxious verbalizations; coat resembling butcher’s attire; visible scalpels
13 years	Essential or well-structured drawings, few colors	Persistent presence of closed doors; oversized light	Rigid, frightened, or absent patients	Sharp instruments (scissors); verbalizations of fear

**Table 2:** Recurrent symbols in the drawings and their clinical–emotional meaning.

Symbol	Description	Clinical–Emotional Meaning
Enlarged dental-unit lamp	Central, very large element	Invasiveness, threat, vulnerability
Disproportionate/sharp instruments	Syringes, drills, pliers resembling weapons	Fear of pain, projected aggressiveness
Closed doors/windows	Enclosed environment with no escape routes	Constraint, anxiety, loss of control
Objects placed between dentist and patient	Obstacles positioned between figures	Relational distance, need for protection
Patient lying down and rigid	Motionless “operating-table” posture	Passivity, loss of control
Menacing dentist	Altered proportions, harsh features	Dentist perceived as punitive or hostile
Absence of the patient	The child does not depict themselves in the scene	Avoidance, emotional dissociation
Dark colors (Black/Intense Red)	Heavy contours, highlighted areas	Fear, tension, aggressiveness
Poor setting	Few details, almost empty clinic	Low familiarity, focus on perceived threat
Rigid figures/large eyes/spiky hair	Angular strokes, tense postures	Tension, alertness, hypervigilance

## Data Availability

The data supporting the findings of this study are available from the corresponding author upon reasonable request.

## Abbreviations

DFA	Dental Fear and Anxiety
WHO	World Health Organization
